# An Electromyographic Study Comparing Muscle Function During Supination and Pronation of the Forearm

**DOI:** 10.7759/cureus.101255

**Published:** 2026-01-10

**Authors:** Suresh Kondi, Thivagar Murugesan, Neil Postans, Paavana Thumri, Ketan Kantamaneni, Shahbaz Ansari, Simon Pickard

**Affiliations:** 1 Trauma and Orthopedics, Salford Royal Hospital NHS Trust, Salford, GBR; 2 Trauma and Orthopedics, Shrewsbury and Telford NHS Foundation Trust, Shrewsbury, GBR; 3 Trauma and Orthopedics, The Robert Jones and Agnes Hunt Orthopedic Hospital NHS Foundation Trust, Oswestry, GBR; 4 Trauma and Orthopedics, East Kent University Hospitals NHS Foundation Trust, Ashford, GBR; 5 General Internal Medicine, Manchester Royal Infirmary, Manchester, GBR

**Keywords:** electromyography (emg), forearm rotation, muscle activation pattern, pronation, sport biomechanics, supination

## Abstract

Background: Forearm pronation and supination are fundamental movements essential for daily activities and clinical applications. While supinator and biceps brachii contribute to supination, and pronator quadratus and pronator teres facilitate pronation, the precise activation patterns and torque-dependent recruitment strategies of these muscles remain incompletely understood. Conflicting evidence exists regarding muscle contribution at varying load conditions, with implications for rehabilitation protocols, surgical planning, and prosthetic design.

Purpose: This study aimed to quantify and compare the electromyographic (EMG) activity of forearm rotator muscles during isometric contraction under progressively increasing torque loads during both supination and pronation movements.

Methods: Four healthy right-handed subjects (3 males, 1 female; mean age 32.5 years) underwent simultaneous EMG and motion capture recording. Surface electrodes captured activity from biceps brachii, triceps, pronator teres, and pronator quadratus, while fine-wire electrodes measured supinator muscle activity. Participants maintained a neutral forearm position against applied loads of 1 kilogram positioned at increasing distances (10-35 cm) from the supination axis, creating progressively higher torques. EMG signals were filtered, rectified, normalized, and analyzed using root mean square values across three trials per loading condition.

Results: During supination resistance, the supinator demonstrated higher activation at lower torques compared to the biceps brachii. As applied torque increased, the biceps brachii activity increased proportionally more than the supinator, indicating load-dependent recruitment. Triceps showed increased co-activation for joint stability. During pronation resistance, the pronator quadratus exhibited greater activity at lower torques relative to the pronator teres. With increasing torque, the pronator teres demonstrated relatively greater activation increases than the pronator quadratus. Both movement patterns demonstrated progressive recruitment of multi-joint muscles as torque demands increased.

Conclusions: This pilot EMG study (n=4) provides preliminary descriptive evidence suggesting that supinator and pronator quadratus primarily govern low-torque forearm rotation, while biceps brachii and pronator teres become increasingly dominant during high-torque demands. These observed patterns are consistent with a hierarchical muscle recruitment strategy optimized for mechanical efficiency and joint stability. Understanding these activation patterns may have potential clinical applications for rehabilitation protocol design following nerve injury or tendon rupture, surgical planning for nerve transfer procedures, and development of myoelectric prosthetic control algorithms.

## Introduction

Human forearm movements, particularly pronation and supination, are essential for a wide range of activities, from basic daily tasks to complex athletic motions. This movement allows the hand to rotate around its longitudinal axis, enabling the palm to face upward (supination) or downward (pronation). Biceps brachii and supinator are both muscles of the upper limb involved in supination of the forearm [1]. Anatomically, the supinator originates from the lateral epicondyle of the humerus, the radial collateral and annular ligaments, and the supinator crest of the ulna, inserting on the lateral, posterior, and anterior surfaces of the radius [2]. This positioning allows it to directly act on the radius to produce supination. The biceps brachii is a two-headed muscle that plays a dual role as an elbow flexor and a forearm supinator. Its short head originates from the apex of the coracoid process of the scapula, while the long head originates from the supraglenoid tubercle of the scapula [3]. It has its distal tendon inserting on the radial tuberosity and wrapping around the shaft, enabling it to contribute to supination torque. Past research has confirmed that the biceps brachii is not active when the forearm is in the prone position, and electrophysiological studies have demonstrated the action of the biceps brachii when the forearm is supinated, but not when it is pronated [4,5].

With regard to pronation, the two principal muscles are the pronator quadratus and the pronator teres. The pronator quadratus spans the distal anterior aspect of the forearm from the ulna to the radius, while the pronator teres runs obliquely from the medial epicondyle of the humerus and the coronoid process of the ulna to the middle third of the lateral surface of the radius [6]. Their anatomical orientations suggest different mechanical advantages for generating pronation torque under varying conditions, which may explain the conflicting results in the previous literature. It is believed that the supinator initiates supination, with the biceps brachii contributing during fast or resisted supination. Similarly, it is believed that the pronator teres has a greater impact on pronation than the pronator quadratus [7,8].

In recent years, advanced electromyographic (EMG) techniques have allowed more precise quantification of muscle activity. O'Sullivan and Gallwey et al. demonstrated that the relative contribution of the biceps brachii and supinator to supination torque varied not only with torque magnitude but also with elbow flexion angle [9]. Similarly, studies have found that the pronator quadratus shows consistent activation during all pronation activities, while the pronator teres demonstrates more variable recruitment patterns dependent on torque demands [10].

The correlation between muscles during maximum isometric rotational torque remains poorly documented. In terms of torque magnitude, some studies have demonstrated higher supination torque than pronation, while others have reported no difference, and still others have shown higher pronation than supination torque [11-13]. These discrepancies in reported torque are attributed not only to methodological differences but also to variations in testing positions. Recent research by Ikeda et al. found that wrist position substantially affects forearm rotation strength, with extended wrist positions generally producing higher supination torques [14]. The impact of grip type and hand position introduces additional complexity when comparing studies. Marcel-Millet et al. demonstrated that different grip types altered the EMG activity of forearm muscles during isometric contractions, which aligns with the observation that comparisons between studies using different grip techniques are difficult [15].

The clinical relevance of understanding precise muscle function extends beyond orthopedic implant design and rehabilitation studies. Rehabilitation protocols following distal biceps tendon rupture, radial nerve injury, and forearm fractures all benefit from detailed knowledge of compensatory muscle activation patterns. It could also improve our understanding within surgical practice, where the nerve to the supinator may routinely be sacrificed as part of nerve transfer surgery. Furthermore, tendon transfer surgeries, increasingly common in brachial plexus reconstruction, rely on an accurate understanding of muscle function for optimal outcomes. Therefore, this study aimed to quantify and compare the EMG activity of the forearm rotator muscles (supinator, biceps brachii, pronator quadratus, and pronator teres) during supination and pronation efforts against incrementally increasing torque loads. We hypothesized that single-joint muscles (supinator and pronator quadratus) would demonstrate preferential activation at lower torque levels, while multi-joint muscles (biceps brachii and pronator teres) would show increased recruitment at higher torque demands.

## Materials and methods

Subjects

**Figure 1:**
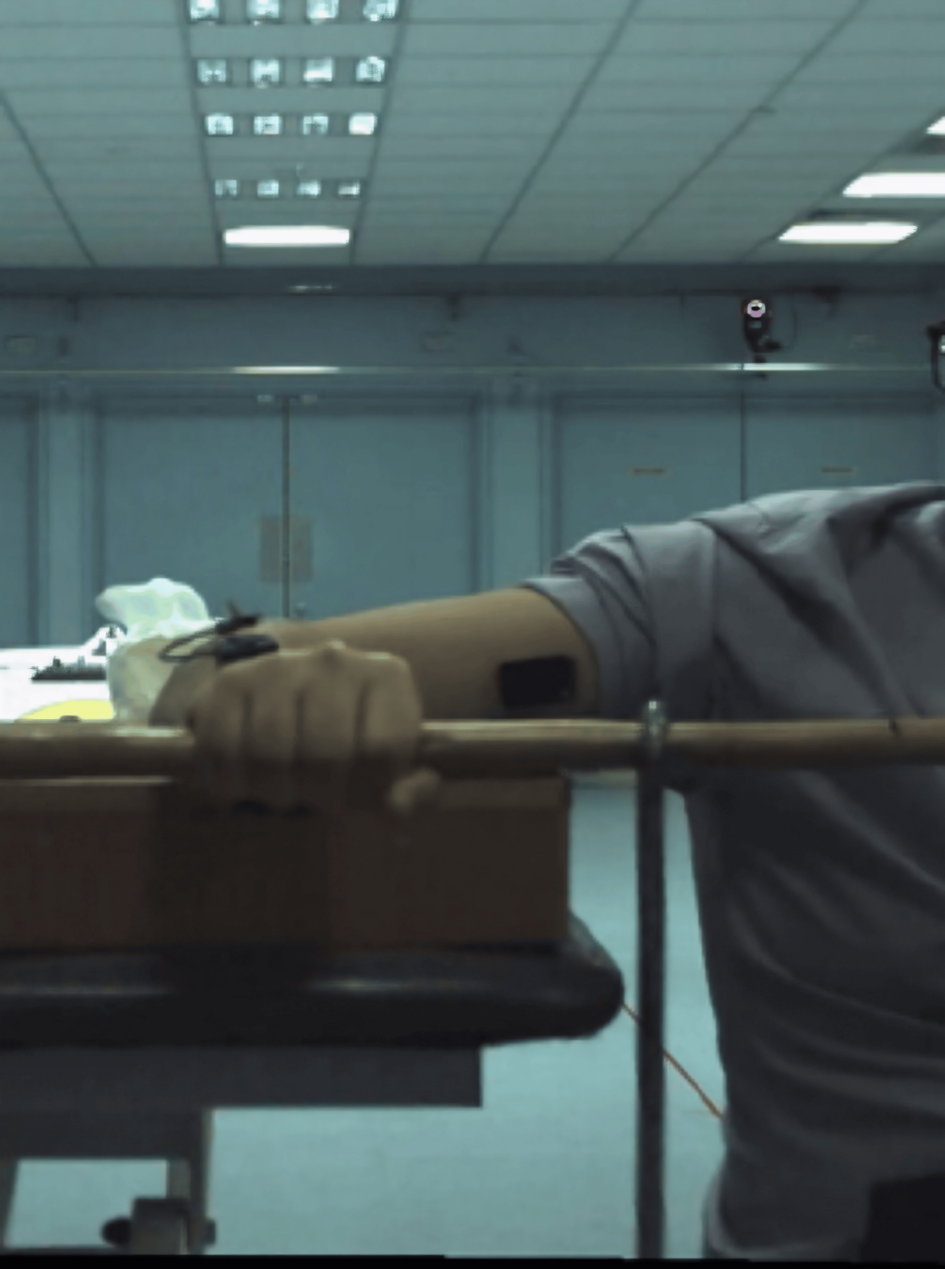
Position of the upper limb during the experiment

Four healthy right-handed subjects (three males and one female) were enrolled in the study. Their mean age was 32.5 years (range 29-39). The participants signed a written consent form allowing EMG testing. This study was approved by the local Research Ethics Board, which follows the Declaration of Helsinki. The shoulder and elbow were supported in a stable position (Figure 1). The Delsys Trigno Inertial Measurement Unit (IMU) EMG system and the Vicon motion capture system were used for simultaneous recording of EMG and video to allow loading phases to be identified. Surface EMG recordings were taken from the biceps brachii, triceps, pronator teres, and pronator quadratus muscles, and fine-wire measurements were taken from the supinator muscle. Electrode placement was guided using an ultrasound probe to confirm muscle position and followed the Surface Electromyography for the Non-Invasive Assessment of Muscles (SENIAM) guidelines [16]. Surface electrode inter-electrode distance was standardized at 20 mm center-to-center, in accordance with SENIAM recommendations. To minimize cross-talk between adjacent muscles, ultrasound guidance was used to visualize muscle boundaries and confirm electrode placement over the target muscle bellies, avoiding tendinous regions and minimizing overlap with neighboring muscles. Fine-wire electrode placement in the supinator muscle was verified through real-time ultrasound visualization during insertion and confirmed via selective muscle activation tests (resisted supination with the elbow extended to minimize biceps contribution). Descriptive statistics were calculated for normalized EMG amplitudes using Microsoft Excel (Microsoft Corporation, Redmond, WA, USA). Mean values and standard deviations (SD) were computed for each muscle across all subjects at each loading condition. The percentage change in muscle activation from baseline (0.98 Nm) to maximum load was calculated to quantify recruitment patterns. Given the small sample size (n=4), results are presented descriptively, with emphasis on observed trends in muscle activation.

An applied load of 1 kilogram was hung from a rod at increasing distances of 10, 15, 20, 25, 30, and 35 centimetres from the supination axis. Participants were instructed to maintain a neutral forearm position during loading so that muscle contraction was isometric. The load was first oriented to apply a pronation torque, to be resisted by the supinator muscles. The load was kept constant for a duration of 3 seconds and repeated three times. EMG was analyzed for these periods, beginning when the investigator released the weight and ending when the investigator gripped the weight again. The same procedure was repeated with the load oriented to apply a supination torque to recruit the pronator muscles.

Surface EMG data were recorded at 1.1 kHz and resampled at 2 kHz for synchronization with the fine-wire EMG, which was sampled at 2 kHz. EMG signals were high-pass filtered to remove motion artefact (2nd-order Butterworth filter with reverse pass and 20 Hz cutoff), then full-wave rectified and low-pass filtered (2nd-order Butterworth filter with reverse pass and 2 Hz cutoff) to produce an enveloped EMG signal. The 2 Hz cutoff frequency was selected based on the sustained, isometric nature of the contractions (3-second holds). While higher cutoff frequencies (4-6 Hz) are common for dynamic tasks, lower cutoff frequencies are appropriate for slow, sustained isometric contractions to reduce high-frequency noise while preserving the slower envelope dynamics characteristic of steady-state muscle activation patterns. This approach balances signal smoothing with preservation of the underlying physiological signal during the isometric protocol. Signal amplitude was normalized to the maximum in each trial.

EMG was then time-normalized to produce 100 data points for each loading phase, and the root mean square (RMS) was calculated for each phase. Finally, the mean RMS across all three trials for each loading phase was calculated for each subject.

Descriptive statistics were calculated for normalized EMG amplitudes using Microsoft Excel. For each muscle at each loading condition, mean values and SD were computed across all subjects. To quantify the magnitude of muscle recruitment with increasing torque demands, the percentage change in normalized RMS activity was calculated from the baseline condition (0.98 Nm, 10 cm moment arm) to the maximum loading condition (3.43 Nm, 35 cm moment arm) for each muscle. Given the small sample size (n=4), results are presented descriptively, with emphasis on observed trends in muscle activation. Descriptive statistics were used to emphasize effect magnitude (percentage changes in activation) and variability (SDs and coefficients of variation).

## Results

The mean normalized RMS values for all muscles during supination and pronation resistance are presented in tables and illustrated in figures.

Supination resistance

During resistance to pronation (supination effort), the activation patterns of the primary supinator muscles and co-contracting muscles are shown in Table 1.

**Table 1:** Normalized RMS muscle activation during supination resistance Values represent mean±SD of normalized EMG amplitude. EMG: electromyography; RMS: root mean square; SD: standard deviation

Moment (Nm)	Supinator	Biceps	Triceps	Pronator quadratus	Pronator teres
0.98	0.455±0.039	0.129±0.051	0.215±0.033	0.315±0.122	0.198±0.125
1.47	0.532±0.043	0.205±0.104	0.276±0.055	0.377±0.192	0.230±0.152
1.96	0.595±0.057	0.294±0.124	0.398±0.096	0.453±0.237	0.271±0.191
2.45	0.669±0.055	0.435±0.130	0.539±0.129	0.525±0.225	0.329±0.268
2.94	0.708±0.080	0.521±0.167	0.648±0.059	0.547±0.291	0.230±0.072
3.43	0.762±0.113	0.703±0.131	0.785±0.105	0.575±0.315	0.233±0.125

At the lowest applied moment (0.98 Nm), the supinator demonstrated higher activation (0.455±0.039) compared to the biceps brachii (0.129±0.051). As the applied moment increased, both muscles showed increased recruitment, with biceps brachii exhibiting a proportionally greater increase (445%) than the supinator (67%). Triceps activity increased by 265%, consistent with co-activation for joint stability.

Pronation resistance

During resistance to supination (pronation effort), the activation patterns of the primary pronator and co-contracting muscles are shown in Table 2.

**Table 2:** Normalized RMS muscle activation during pronation resistance Values represent mean±SD of normalized EMG amplitude. EMG: electromyography; RMS: root mean square; SD: standard deviation

Moment (Nm)	Pronator quadratus	Pronator teres	Biceps	Triceps	Supinator
0.98	0.313±0.110	0.090±0.025	0.284±0.113	0.300±0.182	0.246±0.079
1.47	0.413±0.081	0.147±0.051	0.288±0.117	0.362±0.165	0.219±0.053
1.96	0.515±0.067	0.245±0.118	0.342±0.133	0.437±0.145	0.249±0.055
2.45	0.633±0.054	0.402±0.201	0.429±0.183	0.579±0.135	0.316±0.086
2.94	0.736±0.043	0.503±0.072	0.443±0.210	0.672±0.051	0.310±0.035
3.43	0.789±0.040	0.728±0.097	0.527±0.250	0.811±0.033	0.337±0.017

Pronator quadratus exhibited higher baseline activation (0.313±0.110) than pronator teres (0.090±0.025) at the lowest moment. With increasing applied torque, the pronator teres demonstrated a greater proportional increase (709%) compared to the pronator quadratus (152%), suggesting preferential recruitment of the multi-joint muscle at higher force demands.

**Figure 2:**
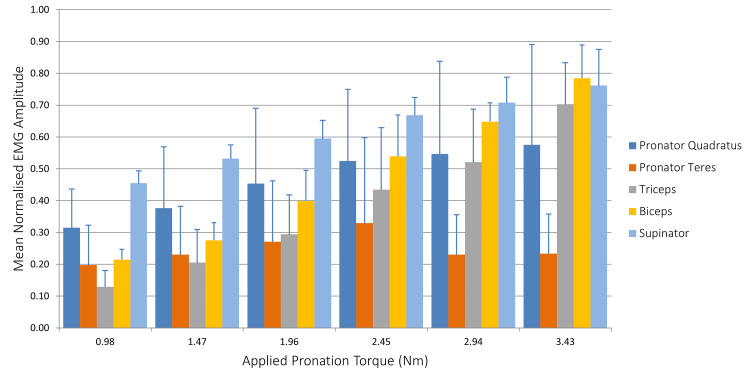
Mean normalized EMG amplitude during applied pronation torque EMG: electromyography

**Figure 3:**
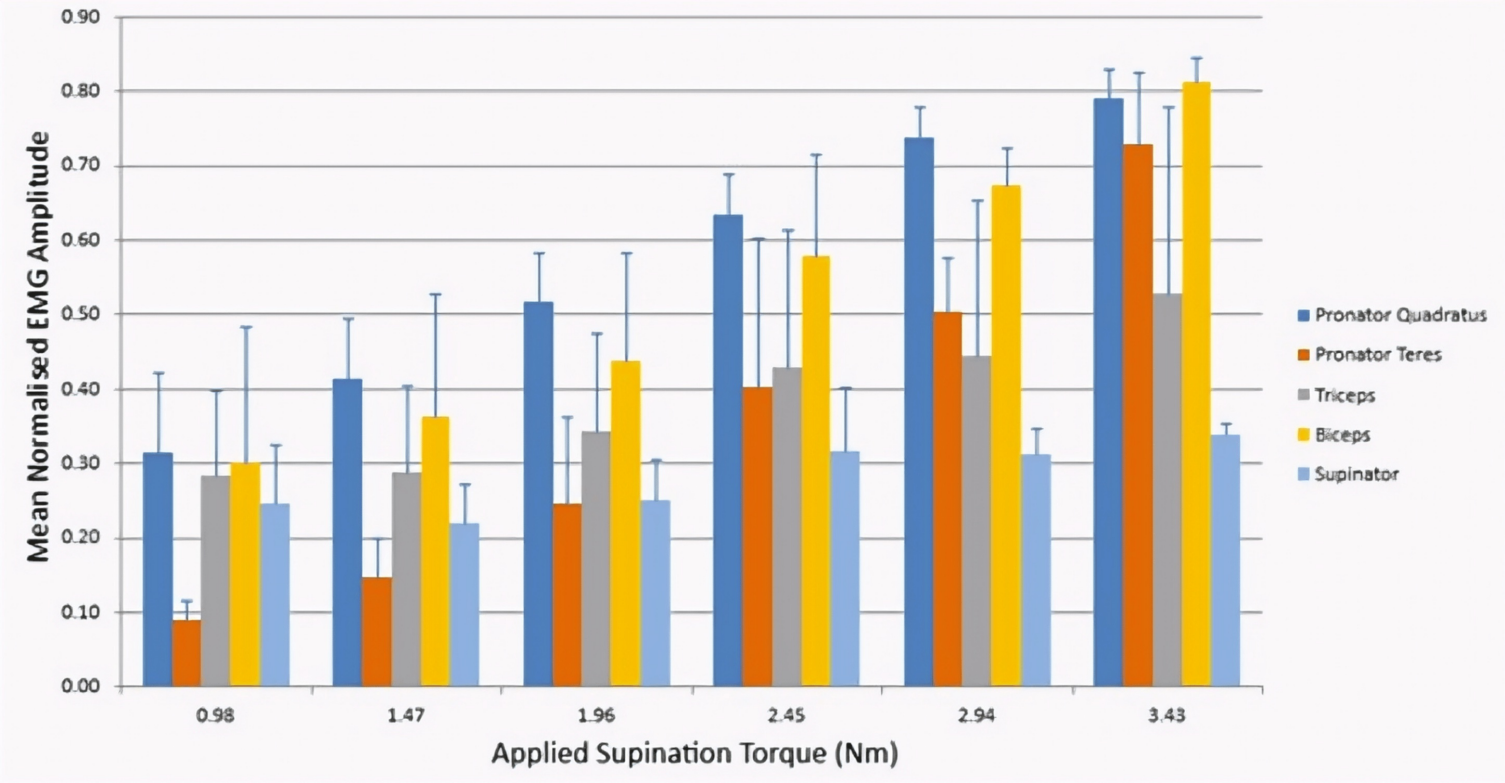
Mean normalized EMG amplitude during applied supination torque EMG: electromyography

The mean RMS of all the muscles in our EMG study for supination and pronation are presented in Figure 2 and Figure 3, respectively. During initial supination, the supinator muscle demonstrated higher firing activity relative to the biceps brachii. There was an increase in recruitment of all muscles as the applied torque was increased, with the exception of the pronator teres. 

The supinator muscle showed increased firing at lower applied torque when compared to other muscles. As the applied torque increased, biceps brachii activity increased relatively more than the supinator. This finding further confirms that the biceps brachii functions not only as a stabilizer but also as a supinator under heavy loading conditions when higher torque is required. Triceps activity also increased due to coactivation for joint stability.

Figure 3 shows the EMG recorded from the same muscles during the applied supination torque. In a pattern similar to that observed during supination, the pronator quadratus muscle showed higher firing activity at lower torque levels relative to the pronator teres. With increasing applied torque, both muscles demonstrated increasing activity, but pronator teres activity increased relatively more than pronator quadratus, suggesting that pronator teres is required in heavier loads, when a higher torque is required.

## Discussion

In our study, with the EMG studies we conducted, we observed that the supinator and pronator quadratus primarily act during low-torque conditions, whereas the biceps brachii and pronator teres play increasingly important roles as torque demands rise. These two muscles, which primarily act at low torque conditions, have a similarity in that they do not cross the joint. This layered activation agrees with foundational EMG principles demonstrated by Naito et al. (1995), showing that the biceps brachii exhibits increased activity during supination and decreased activity during pronation, while the brachialis and brachioradialis display opposite trends. These reciprocal contractions among the flexors allow the biceps brachii to perform supination without inducing elbow flexion [17]. It is important to note that while our EMG findings describe muscle activation patterns, the mechanistic interpretations regarding moment arms, metabolic optimization, and neural strategies are based on theoretical literature and represent plausible explanations that require direct biomechanical testing for confirmation. Our study provides descriptive EMG evidence of differential muscle recruitment patterns, while the biomechanical mechanisms underlying these patterns warrant further investigation.

The transition of activity to the multi-joint muscles at higher loads is consistent with theoretical models of biomechanical leverage, though our EMG data do not directly test the underlying mechanical mechanisms. The effectiveness of a muscle is determined by its moment arm, which is the perpendicular distance from the joint axis to the muscle's line of action. Murray et al. (1995) demonstrated that muscle moment arms at the elbow vary by at least 30% across different elbow and forearm positions, with the biceps showing greater mechanical advantage in extended and supinated positions. This variation helps explain why multi-joint muscles such as the biceps brachii and pronator teres have greater torque-generating capacity than single-joint muscles such as the pronator quadratus and supinator. Therefore, as the required torque exceeds the capacity of the small local muscles, the nervous system must recruit the larger, multi-joint muscles to engage their superior mechanical advantage [18].

Further evidence of positional influence comes from Villalba et al. (2024), who assessed triceps brachii activity during triceps push-downs across different forearm positions. They found that triceps activation increased by 20.6% in the pronated grip compared to the neutral grip, underscoring how forearm rotation modulates muscular engagement and paralleling our findings that position relative to the axis significantly alters EMG activity [19]. Gazzoni et al. (2014) used high-density EMG to quantify forearm muscle activity during wrist and finger movements. They identified that muscles like the flexor digitorum superficialis had higher normalized RMS amplitudes (up to 70%) during finger flexion, indicating the importance of small, deep muscles in fine motor tasks [20]. Similarly, our data suggest that the pronator quadratus, a small, deep muscle, plays a leading role during low-load pronation.

The observed coactivation patterns of antagonist muscles during high-torque conditions align with findings by Solomonow et al. [21] and De Luca and Mambrito [22], who proposed that this coactivation enhances joint stability. This perspective is further supported by work demonstrating that antagonist coactivation strategies are modulated by the task's stability requirements.

Clinical implications

The study’s findings have important implications for both clinical practice and rehabilitation. After distal radius fractures, rehabilitation often focuses on the pronator quadratus muscle, especially since it can be injured during volar plating surgery. These results suggest that starting with gentle, low-torque exercises could effectively target and strengthen the pronator quadratus in the early phases of recovery. As patients regain strength and can tolerate more resistance, exercises that involve greater force would then naturally shift to engaging the pronator teres, providing a logical progression in therapy.

In cases of musculocutaneous nerve injuries (e.g., neuralgic amyotrophy) where biceps function is reduced, understanding the supinator’s contribution during lower-force supination is especially valuable. Rehabilitation can then prioritize supinator-strengthening exercises to compensate for biceps weakness, aligning with strategies described by Kaufman and colleagues for nerve injury recovery [23].

Furthermore, these insights are relevant to the field of prosthetics. Myoelectric control systems, which rely on natural muscle activation patterns, often have difficulty distinguishing between intentions for pronation and supination. By understanding how different muscles are recruited at various force levels, we can help improve the design of prosthetic algorithms for more intuitive and responsive movement.

On a theoretical level, these findings support and refine models of forearm muscle function. Prior work by Prilutsky and Dul et al. has mathematically described how the nervous system distributes workload among muscles to minimize energy use. The observed shift from supinator recruitment at low torque to biceps at higher torque could be an example of metabolic optimization, even though the biceps is not the most mechanically efficient supinator, its larger size may make it preferable for high-force tasks [24].

Finally, the coordination between the biceps and supinator muscles during supination reflects the “dual-control theory” proposed by Latash et al., which suggests that the nervous system simultaneously manages both the movement and joint stabilization. This framework helps resolve prior inconsistencies in muscle coordination literature and guides future research into the complex dynamics of forearm movement [25].

Limitations

The main limitation of this study is the small sample size (n=4). We did not record the activity of additional muscles, such as the wrist flexors and extensors, which previous studies have suggested may play minor roles in forearm supination and pronation. We also did not obtain separate EMG readings for each head of the biceps brachii, which may, in turn, affect overall muscle function. With increasing loads and, therefore, torque, muscle fatigue at different points for each participant may have influenced the results. Additionally, studies incorporating wrist flexors and extensors would provide a more complete picture of forearm muscle synergies, as these muscles have been shown to contribute to forearm rotation in certain contexts.

## Conclusions

This study provides valuable EMG evidence for the functional roles of key forearm muscles during pronation and supination under maximum isometric rotational torque conditions. The findings support the theory that the supinator is the primary muscle for low-torque supination, with the biceps brachii taking on a more stabilizing and dynamic role during high-torque supination of the forearm. Similarly, the pronator quadratus acts as the primary muscle for low-torque pronation, with the pronator teres serving as a stabilizer. These findings should be interpreted as preliminary, given the exploratory nature of this pilot study with four participants. Larger studies with more diverse populations are needed to confirm these patterns and establish their generalizability. Additionally, future research should incorporate direct biomechanical measurements to test the mechanistic hypotheses proposed here: high-torque forearm pronation. Improved understanding of the exact function of these muscles could contribute to biomechanical modelling, rehabilitation protocols following surgery or for musculoskeletal injuries, and prosthetic designs with myoelectric control systems.

## References

[1] Moore KL, Dalley AF, Argur MR (2014). Moore Clinically Orientated Anatomy. https://onlinelibrary.wiley.com/doi/abs/10.1002/ca.22316.

[2] Standring S (2016). Gray's Anatomy: The Anatomical Basis of Clinical Practice. Gray's Anatomy Series 41st edn.: Elsevier Limited.

[3] (2023). Biceps brachii muscle. https://www.kenhub.com/en/library/anatomy/biceps-brachii-muscle.

[4] Basmajian JV, Latif A (1957). Integrated actions and functions of the chief flexors of the elbow: a detailed electromyographic analysis. J Bone Joint Surg Am.

[5] Gordon KD, Pardo RD, Johnson JA, King GJW, Miller TA (2004). Electromyographic activity and strength during maximum isometric pronation and supination efforts in healthy adults. J Orthop Res.

[6] Otoshi K, Kikuchi S, Shishido H, Konno S (2014). The proximal origins of the flexor-pronator muscles and their role in the dynamic stabilization of the elbow joint: an anatomical study. Surg Radiol Anat.

[7] Basmajian JV (1962). Muscles alive. Their functions revealed by electromyography. J Med Educ.

[8] Haugstvedt JR, Berger RA, Berglund LJ (2001). A mechanical study of the moment-forces of the supinators and pronators of the forearm. Acta Orthop Scand.

[9] O'Sullivan LW, Gallwey TJ (2002). Upper-limb surface electro-myography at maximum supination and pronation torques: the effect of elbow and forearm angle. J Electromyogr Kinesiol.

[10] Ibáñez-Gimeno P, Galtés I, Jordana X, Malgosa A, Manyosa J (2014). Biomechanics of forearm rotation: force and efficiency of pronator teres. PLoS One.

[11] Timm WN, O’Driscoll SW, Johnson ME. An KN (1993). Functional comparison of pronation and supination strengths. J Hand Ther.

[12] Winters JM, Kleweno DG (1993). Effect of initial upper-limb alignment on muscle contributions to isometric strength curves. J Biomech.

[13] Kramer JF, Nusca D, Bisbee L, MacDermid J, Kemp D, Boley S (1994). Forearm pronation and supination: reliability of absolute torques and nondominant/dominant ratios. J Hand Ther.

[14] Ikeda K, Kaneoka K, Matsunaga N, Ikumi A, Yamazaki M, Yoshii Y (2025). Effects of forearm rotation on wrist flexor and extensor muscle activities. J Orthop Surg Res.

[15] Marcel-Millet P, Gimenez P, Groslambert A, Ravier G, Grospretre S (2021). The type of visual biofeedback influences maximal handgrip strength and activation strategies. Eur J Appl Physiol.

[16] Hermens HJ, Freriks B, Disselhorst-Klug C, Rau G (2000). Development of recommendations for SEMG sensors and sensor placement procedures. J Electromyogr Kinesiol.

[17] Naito A, Yajima M, Fukamachi H, Ushikoshi K, Sun YJ, Shimizu Y (1995). Electromyographic (EMG) study of the elbow flexors during supination and pronation of the forearm. Tohoku J Exp Med.

[18] Murray WM, Delp SL, Buchanan TS (1995). Variation of muscle moment arms with elbow and forearm position. J Biomech.

[19] Villalba MM, Fujita RA, Junior CI, Gomes MM (2024). Forearm position influences triceps brachii activation during triceps push-down exercise. Int J Strength Cond.

[20] Gazzoni M, Celadon N, Mastrapasqua D, Paleari M, Margaria V, Ariano P (2014). Quantifying forearm muscle activity during wrist and finger movements by means of multi-channel electromyography. PLoS One.

[21] Baratta R, Solomonow M, Zhou BH, Letson D, Chuinard R, D'Ambrosia R (1988). Muscular coactivation. The role of the antagonist musculature in maintaining knee stability. Am J Sports Med.

[22] De Luca CJ, Mambrito B (1987). Voluntary control of motor units in human antagonist muscles: coactivation and reciprocal activation. J Neurophysiol.

[23] Bhat SG, Noonan EJ, Mess G, Miller EJ, Shin AY, Kaufman KR (2023). Characterization of elbow flexion torque after nerve reconstruction of patients with traumatic brachial plexus injury. Clin Biomech (Bristol).

[24] Prilutsky BI, Zatsiorsky VM (2002). Optimization-based models of muscle coordination. Exerc Sport Sci Rev.

[25] Latash ML, Huang X (2015). Neural control of movement stability: lessons from studies of neurological patients. Neuroscience.

